# Cross-Scale Causality and Information Transfer in Simulated Epileptic Seizures

**DOI:** 10.3390/e23050526

**Published:** 2021-04-25

**Authors:** Kajari Gupta, Milan Paluš

**Affiliations:** Department of Complex Systems, Institute of Computer Science of the Czech Academy of Sciences, Pod Vodárenskou Věží 2, 182 07 Prague 8, Czech Republic; gupta@cs.cas.cz

**Keywords:** multiscale dynamics, interactions, Granger causality, information transfer, epilepsy model

## Abstract

An information-theoretic approach for detecting causality and information transfer was applied to phases and amplitudes of oscillatory components related to different time scales and obtained using the wavelet transform from a time series generated by the Epileptor model. Three main time scales and their causal interactions were identified in the simulated epileptic seizures, in agreement with the interactions of the model variables. An approach consisting of wavelet transform, conditional mutual information estimation, and surrogate data testing applied to a single time series generated by the model was demonstrated to be successful in the identification of all directional (causal) interactions between the three different time scales described in the model. Thus, the methodology was prepared for the identification of causal cross-frequency phase–phase and phase–amplitude interactions in experimental and clinical neural data.

## 1. Introduction

Epilepsy is a chronic neurological disorder that occurs in the central nervous system. It is known to affect 0.5% of the general populations in developed countries [1]. In this disorder, the affected area of the brain shows spontaneous and repeated seizure activity lasting for a few minutes. A seizure is an extreme event that occurs in the brain dynamics when a large number of neuron becomes excessively active at the same time and shows higher and synchronized oscillations. These excessive and synchronous activities are believed to be caused by an imbalance between excitation and inhibition within the neuronal population and, as a macroscopic property in brain network, can be observed by an electroencephalogram (EEG) [2]. Understanding the dynamical route to seizures has been a challenge for many years. Researches have shown that different stochastic circumstances in neuronal activity caused by unstable metabolism, systematic intoxication, and documented brain insult can emerge into seizure activity in a normal brain [3]. Many dynamical paths to seizure explanation using the concept of slow permittivity variables and the bistable structure of dynamics [4,5] and domino-like transient dynamics [6] have also been reported. Theoretical models governing nonlinear equations that can show the nature of ictal and normal dynamics have been proposed [7,8]. In these models, the researchers have also shown pre-ictal spikes as a reasonable indicator preceding seizures [9]; however, an actual mechanism of this complex phenomena is still out of reach.

In order to understand seizure activity from a dynamical point of view, the theoretical model *Epileptor* [5], which shows the bistable nature of normal (interictal) and ictal (seizure) states, was developed. Jirsa et al. [5] described the Epileptor model as a five-variable coupled nonlinear dynamical model, where both the “normal” and “ictal” states of brain activity coexist, describing onset, time course, and offset of the ictal-like discharges, including the recurrence of the events. The model consists of one subsystem (subsystem 1) with two state variables, responsible for fast discharges, and a second subsystem (subsystem 2) with two state variables as well, with sharp wave events (SWEs). These are linked by a variable with a very slow time scale called the permittivity variable. In the model, the time scales of the variables play a major role in generating ictal and interictal-like states and their recurrence. We can see that a slower time scale is involved to carry forward the transition between two states and that the seizure genesis involves very fast oscillations [10]. There is active research work to further develop and apply the Epileptor model. For instance, Courtiol et al. [11] extended the model and, using the neuroinformatics platform The Virtual Brain, they demonstrated changes in the brain network’s functional connectivity. Chang et al. [12] studied the seizure-generation process and identified features of transitions between dynamical states, such as critical slowing down; in experimental data; as well as in simulations using the Epileptor model. However, a detailed analysis of how time scales of each variable affects one another in the Epileptor model has not been conducted yet. This motivates us to find the causal relation between the time scales involved for epileptic genesis using the recently developed method of cross-scale causality analysis [13,14].

Any scientific discipline strives to explain the causes of observed phenomena. Thus, causality has been an intensively debated concept in various fields for many years. The mathematical concept of measurable or computable causality is accredited to Granger [15,16], who introduced the idea of causality for predicting a process’s future, with a better accuracy using the past of another process. In this specific definition of causality, known as Granger causality, it is said that a process $X_{t}$ Granger causes another process $Y_{t}$ if the future value of $Y_{t}$ can be better predicted with the information of the past value of $X_{t}$ and $Y_{t}$ instead of only the past value of $Y_{t}$. In the following, we use the term “causality” in the sense of the Granger causality.

Searching for a generalization of the Granger causality concept using information theory, Paluš et al. [17] proposed to measure the information transferred from one system to another using the conditional mutual information (CMI). The conditional mutual information is proposed as an information-theoretic formulation and a nonlinear generalization of the Granger causality. Schreiber [18] introduced a functional of (conditional) probability distribution called transfer entropy. The equivalence of the two functionals was shown by Paluš and Vejmelka [19]. Barnett et al. [20] showed analytically that the transfer entropy is equivalent to Granger causality for Gaussian processes. Therefore, the directed coupling representing a causal influence of one system to another is usually interpreted as information transfer.

In order to study causality between time scales, the concept of cross-scale causality was proposed by Paluš [13,14]. In the case of multiscale systems, CMI is computed from oscillatory components extracted from multiscale time series using the complex continuous wavelet transform (CCWT). The oscillatory components then can be represented by their instantaneous phases and amplitudes. Then, phase–phase, phase–amplitude, and amplitude–amplitude causal interactions can be detected [13,14].

Cross-frequency interactions, in particular, cross-frequency phase–amplitude coupling has recently been observed in electrophysiological signals reflecting brain dynamics. Beyond the synchronization phenomena on particular temporal scales, cross-frequency coupling enriches the cooperative behavior of neuronal networks and apparently plays an important functional role in neuronal computation, communication, and learning [21]. Therefore, the detection of cross-frequency interactions in brain signals became an intensive field of research. However, there are also doubts about the reliability of the used methods [22], suggesting that a part of published results are based on spurious results, i.e., false detections of cross-frequency coupling resulting from signal decomposition methods [23]. Moreover, the majority of studies identify cross-frequency interactions without establishing the direction of interactions. Only a few studies focus on causality in cross-frequency interactions, e.g., [24,25,26]. The Epileptor model is a multiscale system in which variables operating on different time scales are coupled by deterministic equations in a causal way: A time derivative of a particular variable is given as a function of itself and one or more other model variables. Therefore, we can use this model as a generator of time series with known cross-frequency causal interactions. A single time series, resulting from integration of the Epileptor model, underwent an analysis using the approach consisting of wavelet transform, conditional mutual information estimation, and surrogate data testing. The method, described below, was demonstrated to be successful in the identification of all directional (causal) interactions between three different time scales described in the model. Thus, this methodology was prepared for the identification of causal cross-frequency phase–phase and phase–amplitude interactions in experimental and clinical neural data.

## 2. Overview of Methods

### 2.1. Measuring Dependence with Mutual Information

Consider a discrete random variable *X* with a set of values Ξ. The probability distribution function (PDF) for *X* is p(x)= Pr{X=x}, x∈Ξ. We denote the PDF by p(x), rather than $p_{X}\left(x\right)$, for convenience. Analogously, in the case of two discrete random variables *X* and *Y* with the sets of values Ξ and Υ, respectively, their probability distribution functions are denoted as p(x), p(y) and their joint PDF is denoted as p(x,y). The *entropy*H(X) of a single variable, say *X*, is defined as

(1) $$H\left(X\right)=-\sum_{x\in \Xi }p\left(x\right)\log p\left(x\right),$$

and the *joint entropy* H(X,Y) of *X* and *Y* is

(2) $$H\left(X,Y\right)=-\sum_{x\in \Xi }\sum_{y\in \Upsilon }p\left(x,y\right)\log p\left(x,y\right).$$

The *conditional entropy* H(Y|X) of *Y* given *X* is

(3) $$H\left(Y|X\right)=-\sum_{x\in \Xi }\sum_{y\in \Upsilon }p\left(x,y\right)\log p\left(y|x\right).$$

The average amount of common information, contained in the variables *X* and *Y*, is quantified by the *mutual information* I(X;Y), defined as

(4) I(X;Y)=H(X)+H(Y)−H(X,Y).

In order to generalize the mutual information for more variables, let us consider *n* discrete random variables $X_{1},\cdots ,X_{n}$ with values $\left(x_{1},\cdots ,x_{n}\right)\in \Xi_{1}\times \cdots \times \Xi_{n}$, with PDF’s $p(x_{i})$ for individual variables $X_{i}$ and the joint distribution $p(x_{1},\cdots ,x_{n})$. The mutual information $I(X_{1};X_{2};\cdots ;X_{n})$, quantifying the common information in the *n* variables $X_{1},\cdots ,X_{n}$ can be defined as follows [27]:

(5) $$I(X_{1};X_{2};\cdots ;X_{n})=$$

$$H\left(X_{1}\right)+H\left(X_{2}\right)+\cdots +H\left(X_{n}\right)-H\left(X_{1},X_{2},\cdots ,X_{n}\right).$$

In the literature, various terms are used for this information-theoretic functional, e.g., “total correlation” [28], “multivariate constraint” [29], “redundancy” [30], or “multiinformation” [31]. Other authors [32,33,34] defined the mutlivariate mutual information in a different way, and the term “redundancy” has a different meaning in their work.

The conditional mutual information I(X;Y|Z) of the variables *X* and *Y* given the variable *Z* is given as

(6) I(X;Y|Z)=H(X|Z)+H(Y|Z)−H(X,Y|Z).

For *Z* independent of *X* and *Y*, we have

(7) I(X;Y|Z)=I(X;Y).

By simple manipulation, we obtain

(8) I(X;Y|Z)=I(X;Y;Z)−I(X;Z)−I(Y;Z).

Thus the conditional mutual information I(X;Y|Z) characterizes the “net” dependence between *X* and *Y* without a possible influence of another variable, *Z*.

It is possible, however, to define mutual information functionals quantifying common information of groups of variables and various multivariate generalizations of the conditional mutual information; see Reference [35].

All of the information theoretic functionals can be defined for continuous random variables. The sums are substituted by integrals and the PDFs by the probability distribution densities [27,36]. Among the continuous probability distributions, a special role is played by the Gaussian distribution. Let $X_{1},\cdots ,X_{n}$ be an *n*-dimensional normally distributed random variable with a zero mean and an n×n covariance matrix $C=\{c_{ij}\}$. Then (see References [35,36] and the references therein),

(9) $$I_{G}\left(X_{1};\cdots ;X_{n}\right)=\frac{1}{2}\;\sum_{i=1}^{n}\log\left(c_{ii}\right)\;-\;\frac{1}{2}\;\sum_{i=1}^{n}\log\left(\sigma_{i}\right),$$

where $c_{ii}$ are the diagonal elements (variances) and $\sigma_{i}$ are the eigenvalues of the covariance matrix C.

### 2.2. Inference of Causality and Time-Delayed Information Transfer

Paluš and Vejmelka [19] proposed a generalization of Granger causality using time-delayed conditional mutual information (CMI) defined as

$$I\left(X(t);Y(t+\tau )|Y(t)\right)=$$

(10) $$I\left(\left(x(t),x(t-\eta ),\cdots ,x(t-(m-1)\eta )\right);y\left(t+\tau \right)|\left(y(t),y(t-\rho ),\cdots ,y(t-(n-1)\rho )\right)\right),$$

where η and ρ are time lags used for embedding the trajectories X(t) and Y(t), respectively. Formally, Y(t+τ) should also be expanded as y(t+τ),y(t+τ−ρ),⋯,y(t+τ−(n−1)ρ; however, only information about one component y(t+τ) in the τ-future of the system *Y* is used for simplicity. On the other hand, extensive numerical experience [19] suggests that the conditional mutual information in the form

(11) $$I\left(x(t);y(t+\tau )|y(t),y(t-\rho ),\cdots ,y(t-(n-1)\rho )\right)$$

is sufficient to infer the coupling direction between the systems X(t) and Y(t). In fact, the dimensionality of the condition must contain full information about the state of the system Y(t), while single components x(t) and y(t+τ) are able to provide information about the directional coupling between the systems X(t) and Y(t).

### 2.3. Interactions over Time Scales

We consider now a system *X* characterized by time series {x(t)} that evolves simultaneously on many time scales. In order to find the interactions among different time scales, we apply a scale-wise decomposition [37,38,39,40,41], which results in (quasi)oscillatory components $s_{f}\left(t\right)$ distinguished by the scale or frequency *f*. For simplicity, the subscript *f* is omitted in the following text. The (quasi)oscillatory signal (component) s(t) can be conveniently described using the analytic signal approach [42]. For an arbitrary time series s(t), the analytic signal ψ(t) is a complex function of time defined as

(12) $$\psi \left(t\right)=s\left(t\right)+i\hat{s}\left(t\right)=A\left(t\right)e^{i\phi (t)}.$$

The instantaneous phase ϕ(t) of the signal s(t) is then

(13) $$\phi \left(t\right)=\arctan\frac{\hat{s}\left(t\right)}{s(t)},$$

and its instantaneous amplitude is

(14) $$A\left(t\right)=\sqrt{s{\left(t\right)}^{2}+\hat{s}{\left(t\right)}^{2}}.$$

The imaginary part $\hat{s}\left(t\right)$ of the analytic signal ψ(t) can be obtained, e.g., by using the Hilbert transform of s(t) [37,42]. Here, we use the continuous complex wavelet transform (CCWT, hereafter) [43], which can be directly applied to an experimental time series x(t) reflecting the dynamics of a multiscale system. CCWT converts the time series x(t) into a set of complex wavelet coefficients W(t,f):

(15) $$W\left(t,f\right)=\int_{-\infty }^{\infty }\psi \left(t^{\prime }\right)x\left(t-t^{\prime }\right)dt^{\prime }.$$

In the following, we use the complex Morlet wavelet [43]:

(16) $$\psi \left(t\right)=\frac{1}{\sqrt{2\pi \sigma_{t}^{2}}}\exp\left(-\frac{t^{2}}{2\sigma_{t}^{2}}\right)\exp\left(2\pi if_{0}\right),$$

where $\sigma_{t}$ is the bandwidth parameter and $f_{0}$ is the central frequency of the wavelet. $\sigma_{t}$ determines the rate of the decay of the Gauss function, and its reciprocal value $\sigma_{f}=1/\pi \sigma_{t}$ determines the spectral bandwidth. At each time scale given by the central wavelet frequency, the complex wavelet coefficient can be directly used in Equations (13) and (14) for the estimation of the phase ϕ(t) and the amplitude A(t), respectively. We analyze the phase–amplitude cross-scale interaction using Equation (11) as

(17) $$I\left(\phi_{1}\left(t\right));A_{2}\left(t+\tau \right)|A_{2}\left(t\right),A_{2}\left(t-\eta \right),\cdots ,A_{2}\left(t-\left(n-1\right)\eta \right)\right),$$

where $\phi_{1}\left(t\right)$ is the instantaneous phase obtained from the component $W(t,f_{1})$ for the scale given by the wavelet central frequency $f_{1}$ and $A_{2}\left(t\right)$ is the instantaneous amplitude obtained from the component $W(t,f_{2})$ for the scale given by the wavelet central frequency $f_{2}$. In the context of phase–phase interactions, the application of the phase dynamics decreases the dimensionality of the problem—already $I(\phi_{2}\left(t\right);\phi_{1}\left(t+\tau \right)|\phi_{1}\left(t\right))$—when the one-dimensional condition is zero in the uncoupled direction [19]. An even better distinction [19] can be obtained when studying the dependence between the phase of one system, say *i*, and the phase increment $\Delta_{\tau }\phi_{j}\left(t\right)=\phi_{j}\left(t+\tau \right)-\phi_{j}\left(t\right)$ of the second system, say *j*, instead of the dependence between $\phi_{i}\left(t\right)$ and $\phi_{j}\left(t+\tau \right)$, where either i=1 and j=2, or i=2 and j=1, such that we evaluate the conditional mutual information

(18) $$I\left(\phi_{1}\left(t\right);\Delta_{\tau }\phi_{2}\left(t\right)|\phi_{2}\left(t\right)\right)\;\;\mathrm{and}\;\;I\left(\phi_{2}\left(t\right);\Delta_{\tau }\phi_{1}\left(t\right)|\phi_{1}\left(t\right)\right),$$

in a shorter notation $I(\phi_{1};\Delta_{\tau }\phi_{2}|\phi_{2})$ and $I(\phi_{2};\Delta_{\tau }\phi_{1}|\phi_{1})$, respectively. The above-defined conditional mutual nformation functionals are estimated using the equiquantal binning estimator [36,44,45,46,47,48] using eight equiprobable bins in each dimension. The range of the forward time lags τ is discussed in Section 3.2 below. Time series of 65,536 samples entering the computations at sampling times dt=0.1 and dt=0.5 time units were used for the analysis of smaller and larger time scales, respectively.

### 2.4. Statistical Evaluation with Surrogate Data

Estimates of mutual information from finite number of samples are always nonzero, and therefore, it is necessary to relate the CMI values computed from studied data to ranges of CMI values obtained from uncoupled processes that share important properties of the analyzed data. This is the base of the surrogate data testing procedure in which we manipulate the original data in a randomization procedure that preserves the original frequency spectra or variance on all relevant time scales. Here, we use the circular time-shifted surrogates method, which has been shown to be well adapted for causality calculations [49]. For the analyzed time series *X*, we generate 100 independent realizations of time-shifted surrogates as follows: An integer variable *k* is randomly chosen from the interval [0.1,0.9]N, where *N* is the total number of sample points in the series. Then, by moving the first *k* values of X(1),X(2)⋯X(k) to the end of the time series, we generate the circular time-shifted surrogate series $X^{surr}$ as

(19) $$X^{surr}=\left\{X\left(k+1\right),X\left(k+2\right),\cdots ,X\left(N\right),X\left(1\right),X\left(2\right),\cdots ,X\left(k\right)\right\}.$$

The CMI value $I_{d}$ estimated from the studied data is compared with the range of values $I_{s_{i}}$ obtained from a set of surrogate data realizations. Having computed the mean $\bar{I_{s}}$ of the surrogate values $I_{s_{i}}$ and their variance $\sigma_{s}^{2}$, we can define the z-score

(20) $$z=\frac{I_{d}-\bar{I_{s}}}{\sigma_{s}},$$

which is a measure how much the CMI from the analyzed data differs from the range of CMI for the surrogate data. The z-score usually gives a stable estimate from several tens of realizations (30–60), and its value can be related to the statistical significance, provided that the $I_{s_{i}}$ values have a normal distribution.

In the detection of cross-scale interactions in a single time series, performed in this study, the instantaneous phase of the slow component is obtained using the CCWT from the original time series *X* while the phase or amplitude of the faster component is computed from the surrogate versions $X^{surr}$ when computing the surrogate ranges for the evaluated conditional mutual information functionals.

### 2.5. The Epileptor Model

Epileptic electrical activity in the brain, recorded in the form of EEG, also called seizure-like events (SLE), consists of fast electrical discharges, and spike and wave events (SWE). It is observed that these two components operate on different time scales (fast and slow). To ensure an oscillatory fast discharge component, at least two fast state variables ($x_{1},y_{1}$) are necessary, whereas an SWE corresponds to a large amplitude spikes with long-lasting wave components, which requires a slower oscillatory activity, suggesting another pair of slower state variables ($x_{2},y_{2}$). Along with these four state variables, another state variable *z*, slower than SWE, is necessary to ensure the alternating sequence between two components of SLE and to maintain a time-evolving event capturing the properties of SLE. All of these variables are coupled with each other according to the constraints of the bifurcation nature of SLE [5] and gives rise to a five variable model ($x_{1},y_{1},x_{2},y_{2},z$). Essentially, the model captures neuronal discharges due to various ionic current feedback mechanisms related to epilepsy. Hence, the Epileptor model is carried out here using the following six equations adapted from the original five-equation model [5]:

(21) $$\begin{array}{ccc} \dot{x_{1}} & = & y_{1}-f_{1}\left(x_{1},x_{2}\right)-z+I_{rest1}\\ \dot{y_{1}} & = & y_{0}-5{x_{1}}^{2}-y_{1}\\ \dot{z} & = & \left(4\left(x_{1}-x_{0}\right)-z\right)/\tau_{0}\\ \dot{x_{2}} & = & -y_{2}+x_{2}-{x_{2}}^{3}+I_{rest2}+2u-0.3\left(z-3.5\right)\\ \dot{y_{2}} & = & \left(-y_{2}+f_{2}\left(x_{2}\right)\right)/\tau_{2}\\ \dot{u} & = & -\gamma (u-0.1x_{1}) \end{array}$$

where

(22) $$\begin{array}{ccc} f_{1}\left(x_{1},x_{2}\right) & = & {x_{1}}^{3}-3{x_{1}}^{2};\;\left(\mathrm{if}\;x_{1}<0\right)\\ & = & \left(x_{2}-0.6{\left(z-4\right)}^{2}\right)x_{1};\;\left(\mathrm{if}\;x_{1}>=0\right)\\ f_{2}\left(x_{2}\right) & = & 0;\;(\mathrm{if}\;x_{2}<-0.25)\\ & = & 6\left(x_{2}+0.25\right);\;\left(\mathrm{if}\;x_{2}>=-0.25\right) \end{array}$$

and $x_{0}=-1.6$, $y_{0}=1$, $\tau_{0}=2857$, $\tau_{1}=1$, $\tau_{2}=10$, $I_{rest1}=3.1$, $I_{rest2}=0.45$, and γ=0.01. A linear additive noise is introduced to the system by adding a Gaussian white noise with zero mean to subsystems $x_{1},y_{1}$ with variance 0.025 and to subsystem $x_{2},y_{2}$ with variance 0.25. The solution of this stochastic system is obtained by integrating the equations with the Euler–Marayuma method for stochastic differential equations (SDE). The initial conditions were set to $x_{1}=0$, $y_{1}=-5$, z=3, $x_{2}=0$, $y_{2}=0$, and u=0, and the step size for integration dt=0.01 was used. We iterated the system in order to obtain the data range 0–60,000 time units. For the analysis, sampling times dt=0.1 and dt=0.5 were used for the analysis of smaller and larger time scales, respectively.

Three time scales are involved in this model, the majority of equations carry the unit time scale $\tau_{1}=1$ implicitly (it does not appear in the equations). The slow permittivity variable is *z*, with the largest (slowest) time scale $\tau_{0}=2857$. The first subsystem $\left(x_{1},y_{1}\right)$ is responsible for the fast oscillations, and the second subsystem $\left(x_{2},y_{2}\right)$ is responsible for the slow oscillation due to $\tau_{2}=10$. Here, $\tau_{0}>>\tau_{2}>>\tau_{1}$, and the ratio of time scale between the first and the second subsystem is 0.1.

Having integrated this model, the combination of variables ($-x_{1}+x_{2}$) most faithfully resembles the dynamics of the field potential of the electric activity in epilepsy and is often used for the descriptive dynamics for seizure trajectory [5]. Hence, we take this quantity for our analysis of the data. A specific biophysical equivalence for the *z* variable is unknown; however, it corresponds to the slow biophysical processes that occur during a seizure. In the dynamical Equation (21) stable and epileptic dynamics are defined by the parameter $x_{0}$. The dynamics become completely stable without any seizure (ictal) state if $x_{0}<-2$. With $x_{0}=-1.6$, we observe alternating ictal and interictal states.

## 3. Results and Discussion

### 3.1. Analysis of the Epileptor Model

In order to understand the connections of the variables and how the information flows among them in the model, we constructed an adjacency matrix describing the links among the variables. Considering the vector of the variables in the order $\left(x_{1},y_{1},z,x_{2},y_{2},u\right)$, we formed a 6 × 6 adjacency matrix ($A_{ij}$) that has entry 1 at (i,j) if the *i*th variable (node) is influenced by the *j*th variable (node) and 0 otherwise. Hence, from Equations (21) and (22), we can write

(23) $$A_{ij}=\left(\begin{array}{cccccc} 0 & 1 & 1 & 1 & 0 & 0\\ 1 & 0 & 0 & 0 & 0 & 0\\ 1 & 0 & 0 & 0 & 0 & 0\\ 0 & 0 & 1 & 0 & 1 & 1\\ 0 & 0 & 0 & 1 & 0 & 0\\ 1 & 0 & 0 & 0 & 0 & 0 \end{array}\right)$$

**Figure 1:**
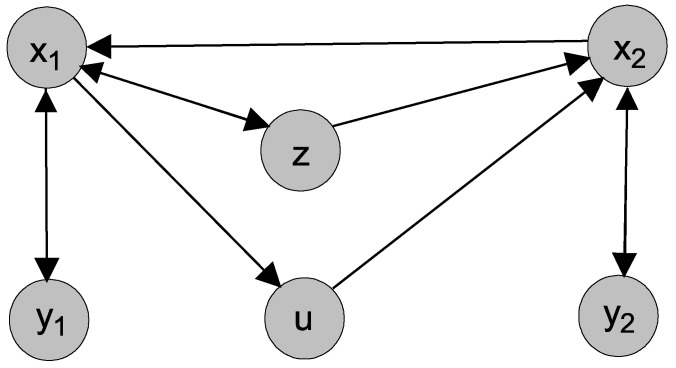
Schematic diagram of the connections of the variables.

As we can see, the adjacency matrix is a directed graph, which is presented in Figure 1. There are pairs that are connected in both directions such as $\left(x_{1},y_{1}\right)$, $\left(x_{2},y_{2}\right)$, and $\left(x_{1},z\right)$. The rest of the pairs are unidirectionally connected. From the equations, we know that $\left(x_{1},y_{1}\right)$ represents the fast subsystem ($\tau_{1}$) and that $\left(x_{2},y_{2}\right)$ represents the slower subsystem ($\tau_{2}$). Now, we want to understand the possible causal information flows among the time scales in the system. When we look at the largest/slowest time scale ($\tau_{0}$), represented by the slow permittivity variable *z*, it is connected unidirectionally to $x_{2}$ ($\tau_{0}\to \tau_{2}$) and $x_{2}$ is unidirectionally connected to $x_{1}$ ($\tau_{2}\to \tau_{1}$). The slow variable *z* is bidirectionally connected to $x_{1}$, suggesting certain information flow from *z* to $x_{1}$ and back ($\tau_{0}\to \tau_{1}$, $\tau_{1}\to \tau_{0}$). We can also see links from $x_{1}$ to *u* and from *u* to $x_{2}$, which can represent indirect links $\tau_{1}\to \tau_{2}$, while the direct link $\tau_{2}\to \tau_{1}$ is reported above. Note that the evolution of *z* and *u* is linear and decaying in their intrinsic dynamics, whereas $\left(x_{1},y_{1}\right)$ and $\left(x_{2},y_{2}\right)$ are nonlinear systems and disturbed with additive noise in this model.

### 3.2. Analysis of Time Series Generated by the Epileptor Model

**Figure 2:**
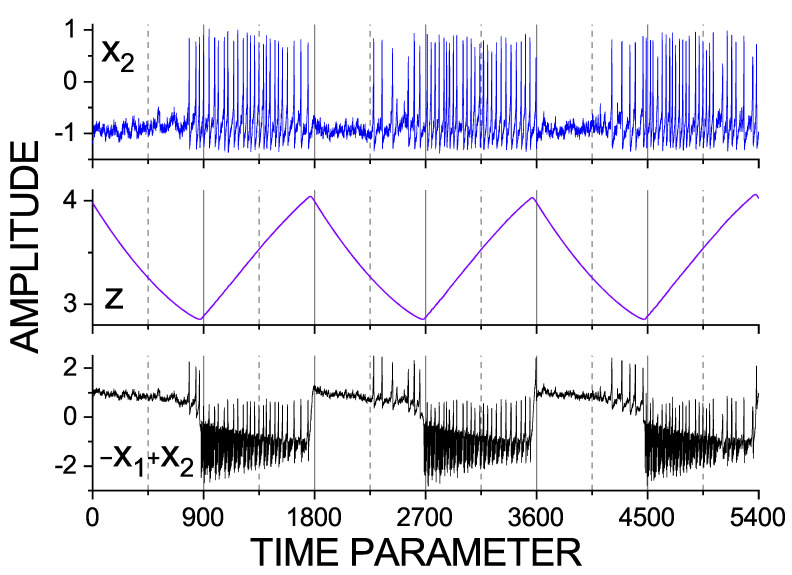
A segment of the time series generated by the Epileptor model (21), from top: variable $x_{2}$ (blue, **top**), *z* (violet, **middle**) and $-x_{1}+x_{2}$ (black, **bottom**). The latter is considered the brain signal (simulated EEG) and analyzed in this study. Note the largest scale in the data: seizures are alternated with interseizure intervals, each lasting about 900 time units.

**Figure 3:**
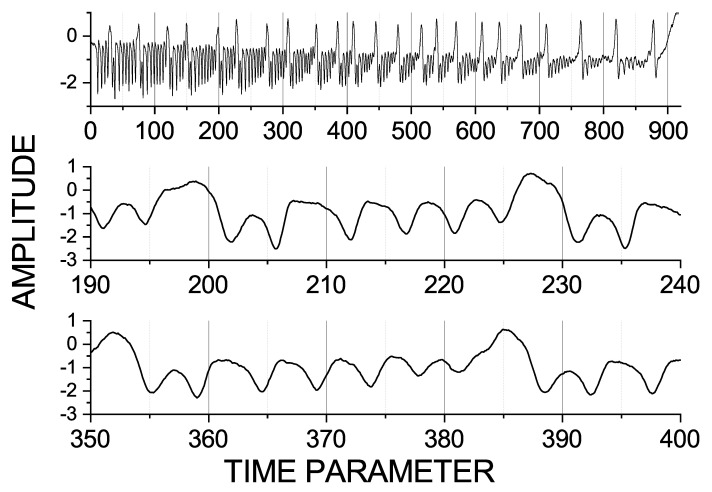
Zoomed views at the variable $-x_{1}+x_{2}$. Note the basic scales in the data: 900, 30, and 5 time units (approximately).

In this section, we discuss the analysis of the data generated by the Epileptor model in order to observe how the time scales involved and their interactions can be detected using the data. As we described above, from the Equation (21) of the Epileptor model, we can identify three time scales involved, denoted as $\tau_{1}=1$, $\tau_{2}=10$, and $\tau_{0}=2857$. Since the time scale parameters are chosen in the denominator, $\tau_{0}=2857$ stands for the largest–slowest time scales in the model, which are reflected in the evolution of variable *z*. Similarly, $\tau_{2}=10$ is reflected in the second subsystem $x_{2},y_{2}$. The variables $x_{1}$ and $y_{1}$ of the first subsystem carry the scale $\tau_{1}=1$. The data are presented in Figure 2 and Figure 3. Figure 2 illustrates a segment of three variables of the model. It is clearly visible that the largest time scale or the slowest frequency corresponds to the period of 900 time units. This shows how seizures (ictal) and inter-seizure (interictal) intervals interchange. We can also understand that this slowest time scale corresponds to the time scale of variable *z* (the middle graph of Figure 2).

In Figure 3, we present the variable $-x_{1}+x_{2}$, chosen for the analysis, using its zoomed versions in order to see more details and shorter scales. Here, we observe a 30 time unit scale, in which we see components of the seizures. The smallest scale, i.e., the period with the fastest oscillation, is about 5 time units. These observed three distinct time scales (900, 30, and 5) reflect the fact that there are three time scales in the Epileptor model itself. Remember though that the variables of the model interact with each other, either unidirectionally or both ways, and that this intricate structure creates the complexity in the model. In this regard, a direct translation from the time scales observed in the equation to the dominant frequencies in the data is not possible, which is why the three dominant time scales seen in the $\left(-x_{1}+x_{2}\right)$ data could be different, both in the in ratios and the absolute values, from the time scales in the equations.

**Figure 4:**
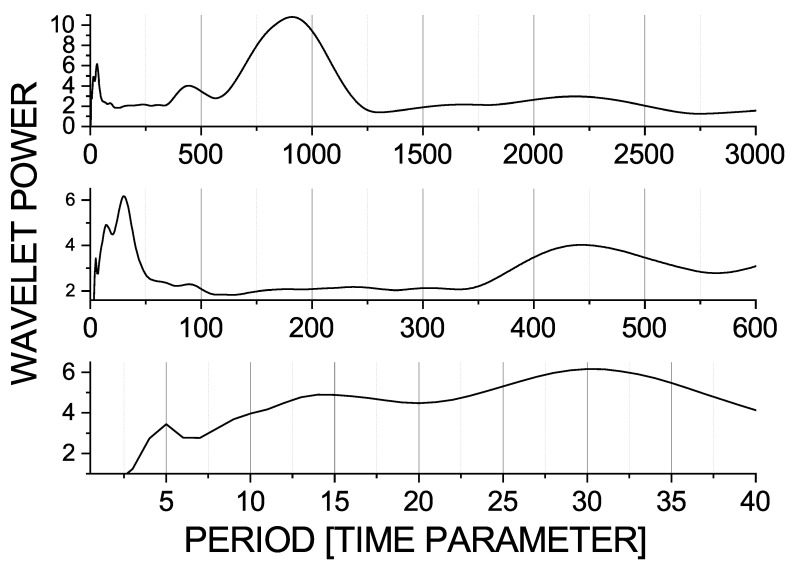
Wavelet power spectra for the variable $-x_{1}+x_{2}$ in the seizure mode as functions of scale (period). Whole scale range (**top**), zoomed range 0–600 (**middle**), and zoomed range 0–40 time units (**bottom**). Besides the basic scales in the data—900, 30, and 5 time units—we can see harmonics, e.g., 15, 90, and 450 time units.

Here, it is also important to note that these time scales are approximates. One segment of a seizure contains one large wave and around five to six small waves. The middle and the bottom graphs of Figure 3 show two different segments of the data in order to demonstrate the variability of the short scales 30 and 5. In the middle graph, we can see two successive large waves starting from the positions 195 and 225, respectively. The first large wave is followed by five small waves. In the bottom figure, the large wave is followed by six small waves and their periods change. Some waves are smaller and some are larger than 5 time units, and the whole pattern is slightly longer than 30 time units. This behaviour can be seen in the time-averaged spectrum in Figure 4 and in its zoomed versions, depicted in order to observe small scales or higher frequencies. Here, we can see that the largest scale, 900, also has a higher harmonic with the period of 450 time units; that the shorter scale, 30 time units, has a higher harmonic for the period of 15 time units; and that both of these peaks are broadbrand. The scale 5 has a broad peak, which reflects the fact that this frequency fluctuates around the mean period of 5 time units.

**Figure 5:**
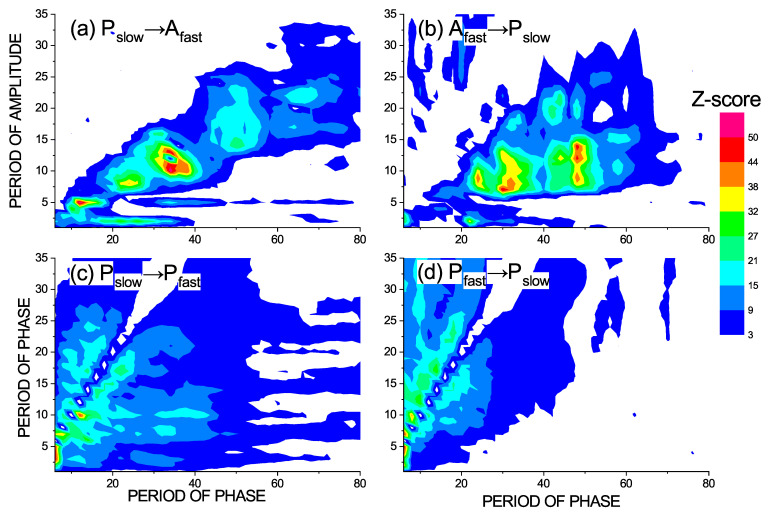
(**a**) The causal influence of the phase of slower oscillations on the amplitude of faster oscillations, i.e., the cross-scale phase–amplitude causality measured by $I\left(\phi_{1}\left(t\right);A_{2}\left(t+\tau \right)|A_{2}\left(t\right),A_{2}\left(t-\eta \right),A_{2}\left(t-2\eta \right)\right)$ and (**b**) the causality in the opposite direction measured by $I\left(A_{2}\left(t\right);\phi_{1}\left(t+\tau \right)|\phi_{1}\left(t\right),\phi_{1}\left(t-\eta \right),\phi_{1}\left(t-2\eta \right)\right)$. (**c**) The causal influence of the phase of slower oscillations on the phase of faster oscillations, i.e., the cross-scale phase–phase causality measured by $I(\phi_{1}\left(t\right);\Delta_{\tau }\phi_{2}\left(t\right)|\phi_{2}\left(t\right))$; and (**d**) the cross-scale phase–phase causality in the opposite direction measured by $I(\phi_{2}\left(t\right);\Delta_{\tau }\phi_{1}\left(t\right)|\phi_{1}\left(t\right))$. The interactions of the small scales 6–80 t.u. with the smallest scales 2–35 t.u. is considered. The areas of significant causality for the z-score z>3 are colored.

The time series of the variable $-x_{1}+x_{2}$ in the seizure mode, i.e., after removal of the interictal segments, was decomposed using the CCWT into a range of time scales, was characterized by by their instantaneous phases (13) and amplitudes (14), was used to estimate the phase–phase causality (18) and phase–amplitude causality (17). Statistical significance of this causality measures is characterized by the *z* score value from Equation (20) and is considered significant when z>3. The significant *z* score is plotted as a color-coded plot between various scales. The cross-scale causal interactions between shorter scales are presented in Figure 5. We can see in Figure 5a the causal influence of the phase of the scales given on the x-axis while the period of the influenced amplitude is on the y-axis (Equation (17)). It shows a pattern of a diagonal-like character, with the ratios 3–5 to 1 representing the finding that, in this direction, there is an influence from the slow-scale phase to fast-scale (approximately three to five times faster) amplitude. Similarly, in Figure 5b, we see the causality in the opposite direction showing a similar pattern, suggesting that there are mutual interactions in these scale ranges. This is a behavior we can also observe in other deterministic models, or models with strong deterministic components (e.g., [50]). It is interesting to note that, in Figure 5a, the influence of the phase of the slow scales (from 30 time units to as high as 80 time units) has a significant influence on the short scale 5 time units and that this influence cannot be observed in the other direction in Figure 5b, suggesting that this causal information flow is unidirectional. In the case of the phase to phase causality in Figure 5c, we see that the phases are mutually connected within a large range of scales. The scale 5 and the harmonics 10 and 15 are influenced unidirectionally by much lower scales ranging from 60 to 80 time units, since there is no influence in the opposite direction (Figure 5d). The large blue region in both the directions in Figure 5c,d suggests that the phases are mutually connected in the large diagonally oriented area. However, exactly on the two diagonals, the white spots suggest that there is no interaction here as the conditional mutual information of the same variables ($\phi_{1}\left(t\right)=\phi_{2}\left(t\right)$) is zero by definition.

**Figure 6:**
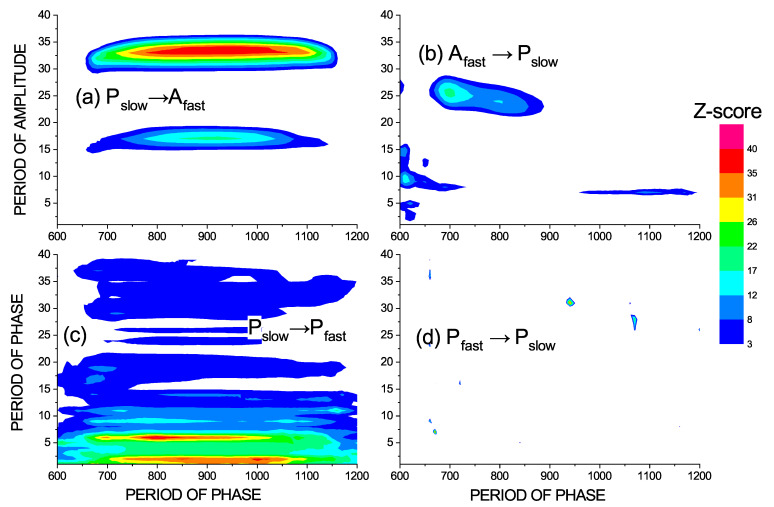
(**a**) The causal influence of the phase of slower oscillations on the amplitude of faster oscillations, i.e., the cross-scale phase–amplitude causality measured by $I\left(\phi_{1}\left(t\right);A_{2}\left(t+\tau \right)|A_{2}\left(t\right),A_{2}\left(t-\eta \right),A_{2}\left(t-2\eta \right)\right)$ and (**b**) the causality in the opposite direction measured by $I\left(A_{2}\left(t\right);\phi_{1}\left(t+\tau \right)|\phi_{1}\left(t\right),\phi_{1}\left(t-\eta \right),\phi_{1}\left(t-2\eta \right)\right)$. (**c**) The causal influence of the phases of slower oscillations on the phase of faster oscillations, i.e., the cross-scale phase–phase causality measured by $I(\phi_{1}\left(t\right);\Delta_{\tau }\phi_{2}\left(t\right)|\phi_{2}\left(t\right))$; and (**d**) the cross-scale phase–phase causality in the opposite direction measured by $I(\phi_{2}\left(t\right);\Delta_{\tau }\phi_{1}\left(t\right)|\phi_{1}\left(t\right))$. The interactions of the large scales 600–1200 t.u. with the smallest scales 2–35 t.u. is considered. The areas of significant causality for the z-score z>3 are colored.

Figure 6 shows the causal influence of the large scales from 600 to 1200 t.u. on the short scales. Unlike the previous case, here, we can see more prominent unidirectional causality. Several spots in Figure 6b can be regarded as false positives (see [13,14]), while in Figure 6a, the causal influence of the phase of the large (slow) scales on the amplitude of the scale 15 and the scale between 30 and 35, is clearly unidirectional. In the case of phase to phase causality in Figure 6c,d, the pattern of significance is much more extended and unidirectional. Here, we can see that the phase of large scales or slow oscillations influences the phases of faster oscillation within the bands related to periods of 5 and 30 t.u. and some of their harmonics.

In the search for causality, the conditional mutual information measures the amount of information contained in the present state of variable, say X(t), about the future Y(t+τ) of the variable *Y*τ time units in advance. The “prediction horizon” τ is a parameter of the method. Paluš and Vejmelka [19] show that more robust results are obtained when using an average over a range of τs instead of selecting a single τ value. Thus, we used the range from 0.1 to 10 time unit, with the step 0.1 t.u. (one sample) in the tests in Figure 5 and from 2 to 200 t.u. with the step 2 t.u. in the tests for large scales in Figure 6. Let us analyze the dependence of CMI on the time lag τ for the selected time scales. In addition to the results for the Epileptor variable $-x_{1}+x_{2}$ time series, we present also results for the time-reversed time series.

Paluš et al. showed [51] that the time reversal in causality analysis can help distinguish between a linear transfer of a time-delayed process and nonlinear interactions of dynamical systems. Indeed, they showed that, in linear autoregressive processes with unidirectional causality, when the independent variable X(t) causes the variable Y(t) using a simple linear, time-delayed term cX(t−τ), the causality direction X→Y is reversed after the time reversal into Y→X. On the other hand, nonlinear dynamical systems violate the Granger causality principle that the cause precedes the effect and that the direction of causality is not reversed after the time reversal. The explanation of this paradox is rooted in the time irreversibility of nonlinear/chaotic systems [51].

**Figure 7:**
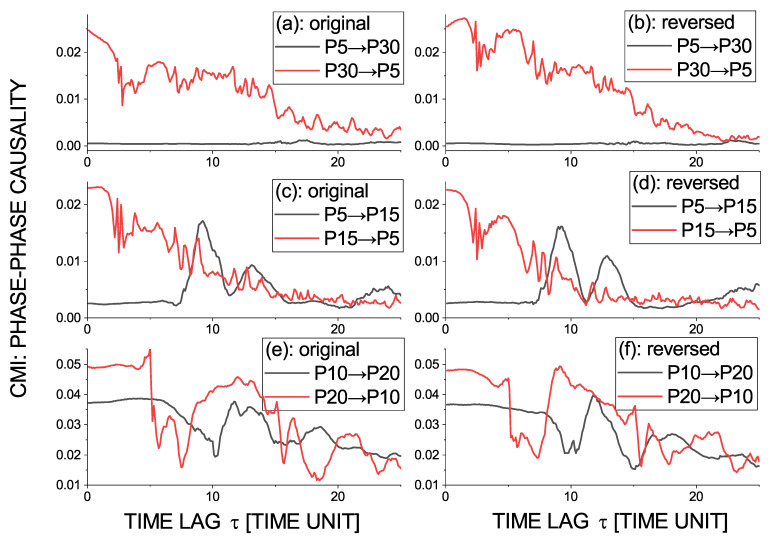
The conditional mutual information (18) as a function of time lag τ, representing the phase–phase (P→P) causality for the selected time scales of the Epileptor variable $-x_{1}+x_{2}$ time series (**a**,**c**,**e**) and for time-reversed time series (**b**,**d**,**f**). Scales and causality directions: (**a**,**b**) P30→P5 (red) and P5→P30 (black), (**c**,**d**) P15→P5 (red) and P5→P15 (black), and (**e**,**f**) P20→P10 (red) and P10→P20 (black).

The conditional mutual information (18) as a function of time lag τ, representing the causal influence of the phase of the oscillatory mode (time scale) with the period of 30 t.u. on the phase of the time scale of 5 time unit (P30→P5), is plotted along the red line in Figure 7a, while the opposite causality (P5→P30) is in the same graph in black. We can see a clear unidirectional causality P30→P5 for a range of τ from the minimum τ=0.1 to more than 20 t.u. After the time reversal (Figure 7b), the character of the causal relation did not change, as we could expect in nonlinear, possibly chaotic systems. Taking the harmonic frequency characterized by the period 15 t.u., we can see (Figure 7c) a similar range of causality P15→P5 as in the case of P30→P5; however, with a time delay of about 7 t.u. the reverse causality P5→P15 emerges. Apparently, the time scale 15 t.u. is a harmonic frequency resulting from the interaction of the basic time scales. Figure 7e illustrates the bidirectional phase–phase causality from the diagonal-like strip in Figure 5c,d, in this case considering the time scales 10 and 20 t.u. All of the presented phase–phase causality relations are invariant with respect to time reversal.

**Figure 8:**
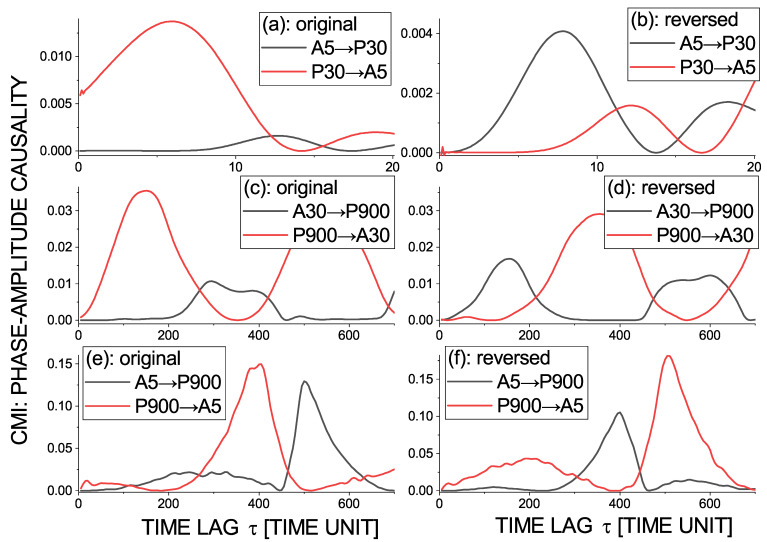
Conditional mutual information (17) as a function of time lag τ representing the phase–amplitude (P→A) causality for the selected time scales of the Epileptor variable $-x_{1}+x_{2}$ time series (**a**,**c**,**e**) and for time-reversed time series (**b**,**d**,**f**). Scales and causality directions: (**a**,**b**) P30→A5 (red) and A5→P30 (black), (**c**,**d**) P900→A30 (red) and A30→P900 (black), and (**e**,**f**) P900→A5 (red) and A5→P900 (black).

The conditional mutual information (17) as a function of time lag τ, representing the causal influence of the phase of the oscillatory mode (time scale) with the period of 30 t.u. on the amplitude of the time scale of 5 t.u. (P30→A5) is plotted in the red line along Figure 8a, while the opposite causality (A5→P30) is in the same graph in black. We can see a unidirectional causality P30→A5 for a range of τ from the minimum τ=0.1 to approximately 10 t.u. After the time reversal (Figure 8b), the direction of the causal relation reverses. We see that the phase–amplitude causality reverses after the time reversal also in other examples below. Thus, the phase of—typically—larger time scales (slower oscillations) causally influences the amplitude of smaller scales (faster oscillations) in a form of a time-delayed linear information transfer. This observation does not mean that the phase–amplitude relation in the epileptic seizures can be described by a simple linear model; however, understanding how this behavior emerges in the nonlinear Epileptor model deserves further study. Additionally, it is interesting to analyze the phase–amplitude relations in real electrophysiological data in this way.

**Figure 9:**
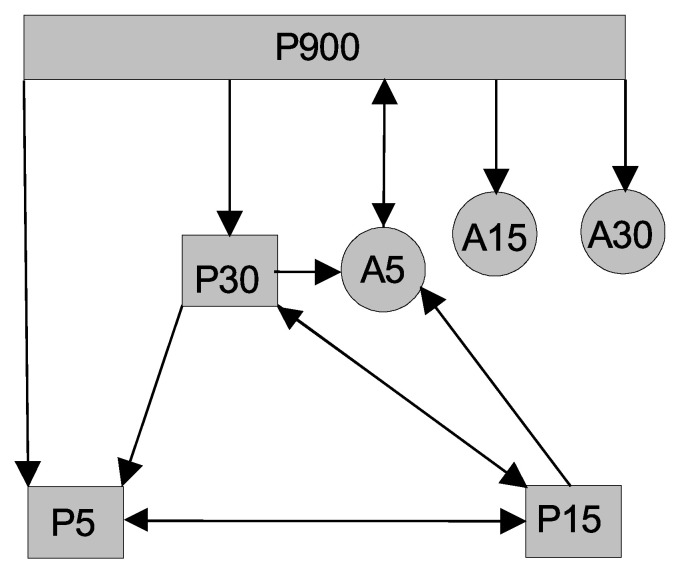
The scheme of all detected causal relations between the main three time scales 5, 30, and 900 t.u. The time scale 15 t.u. of the harmonic frequency is also included. Both phase–phase (P–P) and phase–amplitude (P–A) interactions are considered.

Figure 8c presents the phase–amplitude interaction P900–A30, which is again dominated by the causal influence from larger to smaller scales P900→A30. Finally, in Figure 8e, we can see the phase–amplitude interaction of the largest and smallest scales P900–A5. No significant interaction was detected in Figure 6a,b, and now, we can understand why. There is no significant causal information flow in the time lag range under 200 t.u., used in the tests in Figure 6. Information transfer P900→A5 starts from the time delay τ> 200 t.u. and A5→P900 for τ> 400 t.u. Here, we can see that an appropriate time lag range to detect the cross-scale causality for a large range of scales in a nontrivial problem is set up. Nevertheless, considering the result from Figure 8e, we can summarize our causality detections in a scheme presented in Figure 9.

**Figure 10:**
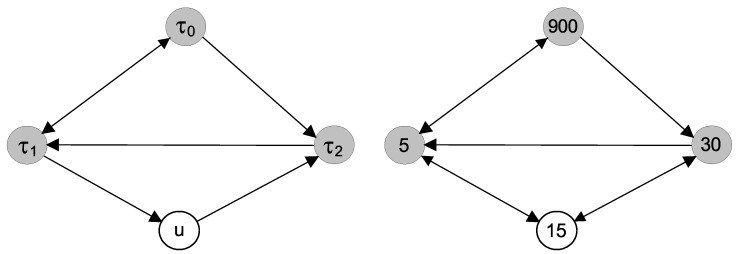
The summary scheme of all directed cross-scale interactions derived from the Epileptor model (21), (22) [**left**] and all causal relations between the main three time scales 5, 30, and 900 t.u. detected from time series of the variable $-x_{1}+x_{2}$ [**right**].

Now, we can return to the scheme in Figure 1, depicting the connections of the Epileptor model variables. Considering the time scales that individual variables bear, we draw the conections between the three time scales $\tau_{0}$, $\tau_{1}$, and $\tau_{2}$ in the left scheme in Figure 10. For completeness, we also illustrate the indirect connection from $\tau_{1}$ to $\tau_{2}$ through the variable *u*. In a similar way, neglecting the difference between the phases and amplitudes, we summarize in the right scheme in Figure 10 the connections between the three main scales 5, 30, and 900 t.u. detected in the time series $-x_{1}+x_{2}$ obtained by the integration of the Epileptor model (21), (22). We can see that we were able to detect all of the direct causal connections between the time scales as they can be derived from the model. For completeness, we also illustrate the indirect connections between the scale 5 and 30 through the scale 15, related to the harmonic frequency. Note, however, that there is no correspondence of the variable *u* and the harmonic scale 15 t.u.

## 4. Conclusions

The approach for detection of cross-scale causality [13,14] was used to uncover cross-frequency causal interactions in modelled epileptic seizures. Conditional mutual information (also known as transfer entropy) was applied to oscillatory components, extracted from a multiscale time series, using complex continuous wavelet transform. The analyzed time series was obtained by integration of the model Epileptor [5] in a state producing seizure activity alternated by quiet periods. The extracted oscillatory components are described using their instantaneous phases and amplitudes; therefore, two types of cross-frequency interactions were studied, namely phase–phase and phase–amplitude causality. Three main time scales as well as their interactions were identified in the data and were in agreement with the interactions of the model variables. The methodology [13,14], which combines wavelet analysis and tools from information theory, was already demonstrated to be useful in the detection of cross-scale causality in climate-related data [13,50], and in this study, it was proven successful in uncovering cross-frequency causality in neural, albeit simulated, data. The next step will be an analysis of the experimental data from either in vitro or in vivo animal epilepsy models and, finally, human electroencephalographic data. We believe that the identification of cross-scale interactions can help better understand the dynamics and genesis of epileptic seizures and can find its use in clinical diagnostics.

## Data Availability

The study used simulated data obtained by integrating the model (21, 22).
